# An Allometric Modelling Approach to Identify the Optimal Body Shape Associated with, and Differences between Brazilian and Peruvian Youth Motor Performance

**DOI:** 10.1371/journal.pone.0149493

**Published:** 2016-03-03

**Authors:** Simonete Silva, Alcibíades Bustamante, Alan Nevill, Peter T. Katzmarzyk, Duarte Freitas, António Prista, José Maia

**Affiliations:** 1 Department of Physical Education, University Regional of Cariri, Crato, Ceará, Brazil; 2 National University of Education Enrique Guzmán e Valle, Lima, Peru; 3 School of Sports, Performing, Arts and Leisure, University of Wolverhampton, Walsall, United Kingdom; 4 Pennington Biomedical Research Center, Louisiana State University, Baton Rouge, Louisiana, United States of America; 5 Department of Physical Education and Sport, University of Madeira, Funchal, Portugal; 6 Faculty of Physical Education and Sports, Pedagogical University, Maputo, Mozambique; 7 CIFI^2^D, Kinanthropometry Lab, Faculty of Sport, University of Porto, Porto, Portugal; Institute of Preventive Medicine, DENMARK

## Abstract

Children from developed and developing countries differ in their body size and shape due to marked differences across their life history caused by social, economic and cultural differences which are also linked to their motor performance (MP). We used allometric models to identify size/shape characteristics associated with MP tests between Brazilian and Peruvian schoolchildren. A total of 4,560 subjects, 2,385 girls and 2,175 boys aged 9–15 years were studied. Height and weight were measured; biological maturation was estimated with the maturity offset technique; MP measures included the 12 minute run (12MR), handgrip strength (HG), standing long jump (SLJ) and the shuttle run speed (SR) tests; physical activity (PA) was assessed using the Baecke questionnaire. A multiplicative allometric model was adopted to adjust for body size differences across countries. Reciprocal ponderal index (RPI) was found to be the most suitable body shape indicator associated with the 12MR, SLJ, HG and SR performance. A positive maturation offset parameter was also associated with a better performance in SLJ, HG and SR tests. Sex differences were found in all motor tests. Brazilian youth showed better scores in MP than their Peruvian peers, even when controlling for their body size differences The current study identified the key body size associated with four body mass-dependent MP tests. Biological maturation and PA were associated with strength and motor performance. Sex differences were found in all motor tests, as well as across countries favoring Brazilian children even when accounting for their body size/shape differences.

## Introduction

Human growth and motor performance (MP) are characterized by their extraordinary plasticity expressed in individual and population variation around the world [1]. Human growth and MP processes are also viewed as endpoints of exposures to different environmental stimuli, namely geo-climatic, socioeconomic and cultural that shape the expression of individual genetic potential [2]. Further, Eveleth and Tanner [3], Tanner [4] and Beunen et al [5] suggested that associations between socioeconomic status (SES) and ethnic background can twist the “picture” of human growth expressed by the 50^th^ percentile, and that a social gradient in growth velocity parameters, biological maturation and attained size are likely to occur.

Motor performance during the pubertal years is undoubtedly linked to body size and shape differences [6]. Additionally, differences in body size and shape are evident in children from developed and developing countries due to marked differences in their way of living induced by social, economic and cultural discrepancies [7]. It is expected that short children with low muscle mass and low SES will be limited in their MP [8]. For example, Chowdhury et al. [9] reported that 5–12 year old Santal children’s MP approximated the 1^st^ percentile of normative data; marked SES gradients were also observed in children from the same region of India.

As variation in body size and maturity status affects MP in growing children [10,11], it is of relevance to normalize or adjust their performance for the possible confounding effect of body size to more adequately interpret their differences [6]. Allometric techniques properly address the effects of age and sex differences in growth and biological maturation in motor performance interpretation [12]. For example, Benefice, Fouére and Malina [13] reported that after controlling for the effects of age and body size, MP differences tended to disappear in Senegalese children of varying degrees of malnutrition and gross motor coordination.

Allometric models have a long history in physical anthropology [14] and biology [15,16], and a recent call has been made for their consideration within molecular biology and gene expression investigations because human size, and what it entails, will always be of concern [17]. These models have been systematically used to partition out differences in size in efficient and elegant ways [18,19], such as differences in VO_2_ peak [20,21], ventricular mass [22], indoor rowing [23], and in a variety of motor tests [6].

Children from different populations differ in their body size and shape due to marked differences in their way of living caused by social, economic and cultural differences [3,24]. The same occurs in their MP [1,12]. As body size and shape may confound human motor performance [25], adjusting physical and MP measures to body size components would allow meaningful individual and group comparisons. Thus, investigating children from countries with different social, demographic and cultural characteristics, such as Brazil and Peru, provides important information for clarifying the correlates of MP. Although allometric models were successfully used in the interpretation of MP in children and youth from developing countries, namely Mozambique [26] and Peru [27], we were not able to find any investigation using this approach with between-country data. Thus, the purpose of this study is to compare Peruvian and Brazilian school children’s MP after normalizing for size descriptors.

## Materials and Methods

### Socio-demographic characteristics of Brazilian and Peruvian samples

The present cross-sectional and cross-cultural study was carried out in Peru (Barranco city) and Brazil (Cariri region). Barranco is one of 43 districts of the province of Lima, capital of the Peru. It is located in the coastal region between the western mountains and the Pacific Ocean and has a subtropical climate. The annual average temperature is 18°Celsius and humidity ranges from 80 to 90%. The district is urbanized in its entirety with a small geographic area and high population density; the primary economy of this city is based on trade and tourism. Cariri is a developing region located in the State of Ceará, in northeast Brazil, comprising 9 cities, of which Juazeiro do Norte, Crato and Barbalha are the most important ones. This region has a low SES when compared to the south, southeast and mid-west regions of Brazil [28]. Moreover, geo-climatic, socioeconomic and cultural characteristics are also very different from other regions [29]. This region has a semi-arid climate with annual temperatures ranging from 20° to 35°Celsius and humidity ranging from 50% (spring) to 80% (winter). Its population became quite heterogeneous due to the high rate of migration from other northeast states, mostly for religious reasons. Geographic, socioeconomic and educational characteristics of Barranco and Cariri are shown in Table 1.

**Table 1:** Geographic, socioeconomic and educational characteristics of Barranco/Peru and Cariri/Brazil.

	Regions	
Demographic characteristics	Barranco (Peru)	Cariri (Brazil)
*Total population*	29984	426742
Official language	Spanish	Portuguese
Total population of school children	16448	93791
Public	8294 (56.9%)^a^	69405 (74.5%)^a^
Private	6271 (43.1%)^a^	24385 (25.5%)^a^
*Geographical characteristics*		
Area (km²)	3.33	1994
Demographic density (persons/km^2^)	9004	385
Human Development Index (HDI)	0.72	0.70
Life expectancy at birth	76	78
Child mortality (per 1000 live births)	12	16
Gross National Income per capita ($US)	465	1498

^a^ Data are frequencies [absolute (n) and relative (%)]

Brazilian data sources: INEP (2013); Atlas Brazil (2014), IBGE (2014), IPECE 2010. Peruvian data sources: INEI (2008); PNUD (2012).

### Study design, sampling procedures and participants

The Peruvian sample comes from the Healthy and Optimistic Growth Study [30] which investigates growth, development and health of children, adolescents and their families. For the present study, 1,410 schoolchildren aged 9 to 15 years were used from the original sample; all were stratified according to sex, area, and school. The Brazilian sample is part of the Healthy Growth in Cariri Study [31,32], a simultaneous mixed-longitudinal and cross-sectional study aiming to investigate growth and motor development of Cariri children and youth. Participants were randomly selected from the three main cities of the region—Juazeiro do Norte, Crato and Barbalha. The Brazilian sample comprises 3,150 children, aged 9 to 15 years. The total sample size is 4,560 children, 2,385 girls and 2,175 boys, from public schools (Table 2). After initial political, educational and health contacts, formal permission to conduct each study was asked from school authorities. Written consent from parents and assent from children were obtained after full explanation of the measurement procedures. The ethical committee of the National University of Education Enrique Guzmán y Valle Faculty in Peru, and the Ethics Research Committee of the Medical School of Juazeiro, in Brazil, approved the two projects.

**Table 2:** Sample size by country, sex and age.

Age (years)	Peru (Barranco)		Brazil (Cariri)		Total	
	Girls	Boys	Girls	Boys	Girls	Boys
9	119	76	136	154	255	230
10	85	84	160	131	245	215
11	111	113	224	192	335	305
12	92	119	309	288	401	407
13	69	64	371	287	440	351
14	125	101	267	239	392	340
15	110	142	207	185	317	327
**Total**	711	699	1674	1476	2385	2175

### Anthropometry

All measurements were made according to standardized techniques [33]. In Peru, height was measured with a portable stadiometer (Sanny, Model ES-2060, Brazil) to the nearest 0.1 cm, and body mass was measured to the nearest 0.1 kg using a digital scale (Pesacon, Model IP68, Peru). In Brazil, height was measured with a portable stadiometer (CARDIOMED^®^ Welmy Model 220, Brazil) to the nearest 0.1 cm, and body mass was measured with a digital scale (TANITA^®^ Model 683W, Brazil) to the nearest 0.1 kg. In both Brazil and Peru sitting height was measured with the subject´s head positioned to the Frankfurt plane and sitting on a bench of 40 cm high. BMI was obtained by the usual ratio of body mass to height (kg∙m^-2^) and reciprocal ponderal index (RPI = height · mass^-0.333^).

### Maturity offset

Maturity offset predicts time before or after age at peak height velocity, and is a non-invasive method. Very simply, sex-specific equations were used with age, body mass, height, sitting height and leg length as predictors [34]. The maturity offset equation estimates the distance each subject is from their expected age (in years) of attainment of peak of high velocity (PHV). The value is expressed in years (either + or -) from PHV. Incorporating maturity offset into our models, the use of a non-linear exponential function is capable of modelling the increasing/decreasing rate in the way maturity offset affects motor performance in addition to other confounding variables.

### Motor Performance

Motor performance was assessed with four tests from the EUROFIT battery [35], namely, static (grip strength) and explosive (standing long jump) muscle strength, and speed/agility (shuttle run). Cardiorespiratory endurance was assessed with the 12-minute run/walk from the American Alliance for Health, Physical Education and Recreation (AAHPERD) [36].

The 12 minute run test: in a previously delimited field, schoolchildren in groups of 10–12 (male, female) run/walk the maximum possible distance in 12 minutes.

The standing long jump test: jumping with feet together and without a preparatory run. The maximum jumping distance is recorded. Two trials were given and the best score was recorded as the maximum jump distance in centimeters.

The grip-strength test: the subjects were instructed to squeeze a calibrated hand dynamometer (Takei TKK 5401, Japan) with maximal force. All schoolchildren performed two trials with each hand. The best trial from each hand was recorded in kg and was used to compute the mean muscle strength. The handle length was adjusted to control for variations in hand size.

The shuttle-run test: each subject performs five cycles (round-trip) at maximum speed between two lines separated by five meters (total distance = 50m); this test was conducted in pairs.

### Physical activity

Physical activity (PA) was assessed using the Baecke questionnaire [37]. This questionnaire comprises a total of 16 questions, and maps three PA domains: school/work, leisure-time (PA_le_), and sport participation (PA_sp_). Individual question scores varied from 1 to 5. Baecke’s questionnaire has been consistently used in Portuguese speaking countries, Portugal [38,39] and Brazil [40,41], as well as in Peru [30,42] and its adaptation was done according to the following steps: first a translation from English to Portuguese was done by an English-speaking expert; second, word accuracy and meaning were discussed with questionnaire development specialists from the University of Porto; third, pilot studies were done in order to find out if children/youth clearly understood what was being asked; fourth, to verify the consistency with which children/youth respond reliability studies were done; fifth, Brazilian versions were already available and re-checked with the Portuguese version; sixth, a Peruvian version were also made according to steps 1 to 4. For the purposes of the present study the total PA score was obtained only from the sum of scores of leisure time PA and sport participation. All schoolchildren answered the questionnaires during their physical education classes under the supervision of the teacher of Physical Education who underwent training sessions provided by the research team members.

### Data quality control

Data quality control was assured in three steps. First, a pilot study was conducted in each country to assess proper standardization of measurements. Second, a reliability-in-field procedure was carried out where three to five students every day were randomly selected and re-measured/assessed during data collection. Technical errors of measurement (TEM) for height and body mass, and ANOVA-based intraclass correlations (R) and corresponding 95% confidence intervals (95% CI) were used to estimate test-retest reliability (Table 3). No significant differences between test and retest means were observed (data not shown). The final step consisted of checking for errors in data entry, inconsistent data, and exploratory data analysis in IBM SPSS 21.

**Table 3:** Reliability estimates for anthropometry and physical tests.

Measures	Peru (Barranco)		Brazil (Cariri)	
Anthropometry	TEM^a^	CV*	TEM	CV
Body Mass (kg)	0.88	2.2%	0.20	0.5%
Height (cm)	0.86	0.6%	0.45	0.3%
Sitting height (cm)	0.86	1.2%	0.94	1.3%
Physical fitness	R	95% CI^b^	R	95% CI^b^
12 minute run (min)	0.84	0.81–0.88	0.87	0.72–0.94
Standing Long jump (cm)	0.98	0.97–0.98	0.96	0.93–0.98
Grip-strength (kg)	0.96	0.94–0.97	0.97	0.96–0.98
Shuttle-run (s)	0.88	0.85–0.91	0.87	0.81–0.92

^a^ TEM, Technical error of measurement;

* CV, Coefficient of variation;

^b^ CI, confidence interval.

### Statistical Methods

Descriptive statistics were computed and expressed as means and standard deviations. ANCOVA was used to test for regional differences in anthropometrics, MP and physical activity using chronological age as a covariate. In order to identify the most appropriate body-size and shape characteristics as well as any categorical differences (sex and age) associated with a variety of physical performance variables, we adopted the following multiplicative model with allometric body size components as advocated by Nevill et al. [6] and Bustamante et al. [27]:

$$\text{Y }=\text{ a }\cdot {\text{mass}}^{{\text{k}}_{1}}\cdot \;{\text{height}}^{{\text{k}}_{2}}\cdot \text{ exp}\left(\text{ b }\cdot \text{ maturity}–\text{offset }+\text{ c }\cdot {\text{PA}}_{1\text{e}}\;+\text{ d }\cdot {\text{PA}}_{\text{sp}}\right)\;\cdot \varepsilon ,$$ (1)

where the intercept “a” is allowed to vary for various categorical or group differences within the population, e.g. sex, age (entered as discrete categories 9 to 15 yrs), and country (Peru or Brazil), This model has the advantages of having proportional body size components and the flexibility of a maturity-offset estimate within an exponential term that will ensure that the measure of physical performance (Y) will always remain non-negative irrespective of the subjects’ maturity-offset estimate. Note that ‘ε’, the multiplicative error ratio, also assumes the error will increase in proportion to the physical performance variable Y.

The model (Eq 1) can be linearized with a log transformation. A linear regression analysis on log(Y) can then be used to estimate the unknown parameters of the log transformed model:

$$\text{log}\left(\text{Y}\right)=\text{ log}\left(\text{a}\right)+{\text{ k}}_{1}\cdot \text{log}\left(\text{height}\right)+{\text{k}}_{2}\cdot \text{log}\left(\text{mass}\right)\;+\text{b}\cdot \left(\text{maturity offset}\right)+\text{ c}\cdot {\text{PA}}_{\text{le}}\;+\text{ d}\cdot {\text{PA}}_{\text{sp}}\;+\text{ log}\left(\text{ε}\right).$$ (2)

where k_1_ and k_2_ are the body mass and height exponents associated with the MP dependent variable. Further categorical or group differences within the population, e.g. sex, age (entered as discrete categories 9 to 15 yrs), and country (Peru or Brazil), can be explored by allowing the constant intercept parameter ‘log(a)’ in Eq 2 to vary for each group (by introducing them as fixed factors within the ANCOVA). Effect sizes were also calculated according to usual procedures within the context of two-sample comparisons. The significance level was set at *P*<0.05.

## Results

Table 4 shows the results of the ANCOVAs between Brazilian and Peruvian children. Brazilian boys and girls are shorter and lighter than their Peruvian peers at all ages. Maturity offset indicates that, on average, Brazilian and Peruvian boys are less mature than girls. Brazilian boys and girls outperformed Peruvian children in all MP tests and are more physically active.

**Table 4:** Means (±standard deviation), p-values and effect size for the differences in anthropometry, motor performance and physical activity between Peru (Barranco) and Brazil (Cariri) for boys and girls.

Characteristics	Peru (Barranco)	Brazil (Cariri)			Peru (Barranco)	Brazil (Cariri)		
	Mean ± SD	Mean ± SD	p	Effect size	Mean ± SD	Mean ± SD	p	Effect size
Anthropometry								
Height (cm)	150.3 ± 13.4	145.6 ± 12.8	<0.001	0.18	146.4 ± 9.6	145.9 ± 10.5	<0.001	0.02
Body mass (kg)	48.8 ± 13.4	39.4 ± 11.2	<0.001	0.36	45.7 ± 10.9	40.2 ± 10.4	<0.001	0.25
BMI (kg/m²)	21.2 ± 3.6	18.2 ± 2.8	<0.001	0.42	21.1 ± 3.4	18.6 ± 3.1	<0.001	0.36
Sitting height (cm)	79.6 ± 6.8	73.1 ± 6.3	<0.001	0.44	78.4 ± 5.6	74.2 ± 5.7	<0.001	0.35
RPI (cm/kg^-0.333^)	41.6 ± 2.2	43.1 ± 2.7	<0.001	-0.29	41.3 ± 2.0	42.9 ± 2.0	<0.001	-0.37
Maturity offset	-1.07 ± 1.7	-2.12 ± 1.4	<0.001	0.32	0.11 ± 1.6	-0.27 ± 1.4	<0.001	0.13
Motor performance								
Shuttle-run test (s)	22.4 ± 2.9	21.7 ± 1.5	<0.001	0.15	24.8 ± 2.7	23.2 ± 1.6	<0.001	0.34
Long jump test (cm)	142.9 ± 30.0	153.3 ± 26.5	<0.001	-0.18	120.9 ± 20.5	131.5 ± 20.6	<0.001	-0.25
Grip-strength test (kg)	20.8 ± 8.5	22.5 ± 7.9	<0.001	-0.10	16.9 ± 5.0	20.1 ± 5.7	<0.001	-0.29
12 minute run test (m)	1386.9 ± 364.0	1907.0±311.6	<0.001	-0.61	1320.7±233.9	1633.9 ± 255.7	<0.001	-0.54
Physical activity								
Sports index	2.37 ± 0.12	2.43 ± 0.49	0.002	-0.08	1.99 ± 0.23	2.26 ± 0.47	<0.001	-0.34
Leisure time index	2.98 ± 0.60	3.01 ± 0.58	0.430	-0.03	2.91 ± 0.60	2.78 ± 0.59	<0.001	0.11

The following allometric analyses will not only identify the optimal body size components associated with these MP tests, but they will also be able to report the populations differences in MP having adjusted for the body-size differences in children between the two regions.

### The 12 minute run test

The model relating distance run to the body size components was found to be:

$$\text{Distance }(\text{m})\;=\;a.{\text{ mass}}^{-0.240}\;\cdot \;{\text{height}}^{0.327}$$ (3)

**Fig 1:**
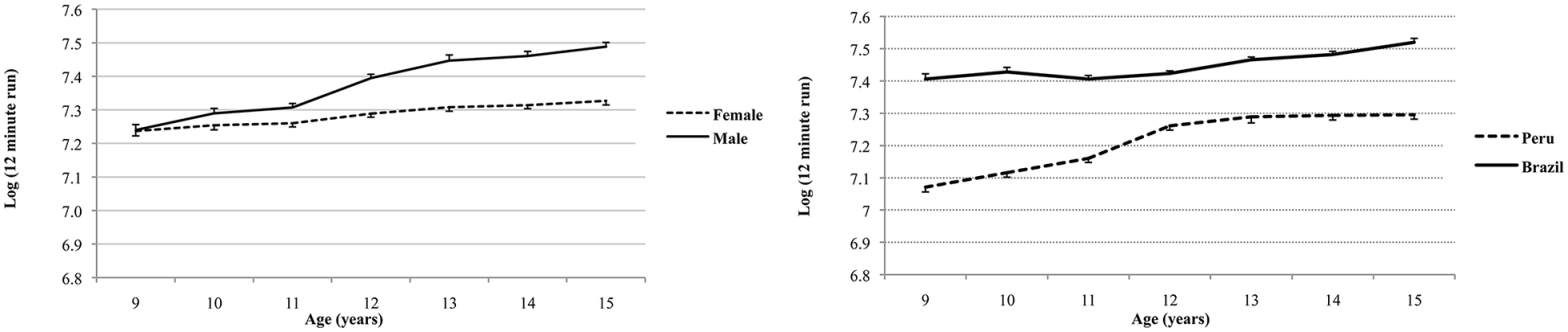
Trends in means (logs of 12 minute run) in boys and girls, irrespective of country, across age (left), and between Peruvians and Brazilians, irrespective of sex, across age (right).

The mass and height exponents were k_1_ = -0.240 [standard error of estimate (SEE = 0.021)] and k_2_ = 0.327 (SEE = 0.084), respectively, but no significant effect due to maturity offset and PA levels (leisure time and sports participation) with no significant maturation offset parameter *b* = 0.017 (SEE = 0.014) and PA *c* = 0.006 (SEE = 0.006) having controlled for differences in mass, height, sex, age and country. Note that the height and mass exponents have opposite signs suggesting that a height-to-weight ratio, not too dissimilar to inverse BMI (reflecting a measure of leanness, see Nevill et al. [43], is the optimal body shape associated with the 12 minute run test. The adjusted coefficient of determination (adj R^2^) was 44.8%. The constant “a” varied significantly with the three main effects of sex, age, and country and all interactions between these categorical variables (p<0.001). From adjusted marginal means, anti-logs were calculated and differences were computed between Peruvians and Brazilians. Brazilian youth run, on average, ~358 m more than Peruvians. Also, from adjusted marginal means, after taking anti-logs, girls run, on average, ~189 m less than boys. Fig 1a shows a marked interaction between “sex” and “age” (p<0.001) favoring boys (p<0.005), especially after 12 years of age. Fig 1b illustrates the interaction between “regions” and “age” with both groups of children and adolescents increasing their running distance by age, but with Peruvians initially rising more steeply but then leveling-off from 14 years onwards.

### The standing long jump test

The multiplicative model relating the standing long jump to body size and maturity offset estimate was found to be:

$$\text{Standing long jump }(\text{cm})\;=\;a.\;{\text{mass}}^{-0.348}\;\cdot \;{\text{height}}^{0.917}\;\cdot \text{ exp}(0.043\;\cdot \text{ maturity offset})\;\cdot \text{ exp}(0.011\;\cdot \;{\text{PA}}_{\text{le}})$$ (4)

**Fig 2:**
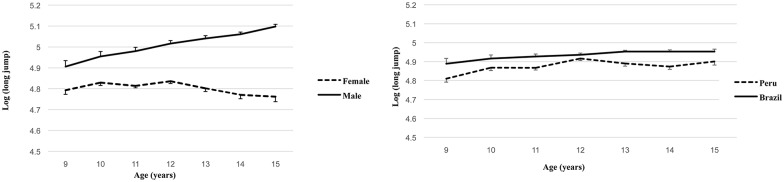
Trends in means (logs of long jump) in all boys and girls, irrespective of country, across age (left), and between Peruvians and Brazilians, irrespective of sex, across age (right).

The mass and height exponents were k_1_ = -0.348[standard error of estimate (SEE = 0.021)] and k_2_ = 0.917 (SEE = 0.097), respectively, with a significant maturation offset parameter b = 0.043 (SEE = 0.010) and PA c = 0.011 (SEE = 0.004). Note that the height-to-mass ratio is almost exactly the reciprocal ponderal index RPI = height · mass^-0.333^. The adj R^2^ was 45.1%, but no significant effect due to sports participation index (p = 0.645) was found having controlled for differences in mass, height, sex, age and country. The constant “a” varied significantly with the main effects of sex, maturity, age, and country and all interactions between these categorical variables (p<0.001). From adjusted marginal means, anti-logs were taken and from them differences were calculated between Peruvians and Brazilians; Brazilian youth jump, on average, ~9.0 cm more than Peruvians. Also, from adjusted marginal means, after taking anti-logs, girls jumps, on average, ~28.0 cm less than boys. Fig 2a shows marked interaction between “sex” and “age” (p<0.001) favoring boys from both countries (p<0.005); girls show a decline in MP from 12 years onwards. Fig 2b illustrates the interaction between “country” (Peruvian or Brazilian) and “age” (9 to 15 years) (p<0.001). Brazilian children increase their jumping performance until 13 years, and then a plateau; on the contrary, Peruvian children show an increase until 12 years and then decrease from 14 to 15 years of age.

### The grip strength test

The multiplicative model relating strength to body size components and maturity offset estimate was found to be:

$$\text{Grip strength }(\text{kg})\;=\;a\;\cdot \;{\text{mass}}^{0.204}\;\cdot \;{\text{height}}^{1.059}\text{ exp}(0.105\;\cdot \text{ maturity offset})\;\cdot \text{ exp}(0.015\;\cdot \;{\text{PA}}_{\text{le}})$$ (5)

**Fig 3:**
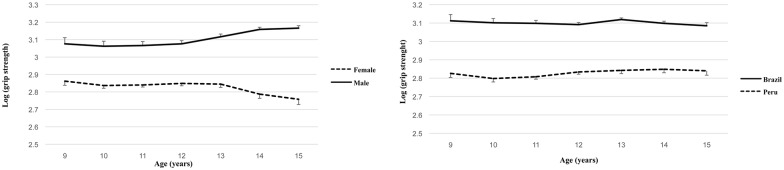
Trends in means (logs of grip strength) in all boys and girls, irrespective of country, across age (left), and between Peruvians and Brazilians, irrespective of sex, across age (right).

The mass and height exponents were k_1_ = 0.204 (SEE = 0.026), and k_2_ = 1.059 (SEE = 0.123), respectively, with a significant maturation offset parameter b = 0.105 (SEE = 0.013) and PA c = 0.015 (SEE = 0.005). The adj R^2^ was 74.5%, but no significant effect due to PA sports participation (p = 0.685) was found having controlled for differences in mass, height, sex, age and country. The constant “a” varied significantly with the main effects sex, maturity, PA, age, and country and all interactions between these categorical variables (p<0.001). From adjusted marginal means, anti-logs were taken, and from them differences were calculated between the two samples. The performance of Brazilian children and youth in grip strength is, on average, 5.3 kg higher than Peruvians. Also, from adjusted marginal means, after taking anti-logs, boys are, on average ~5.4 kg stronger than girls. The interaction between sex, age and country in grip strength (p<0.001) is plotted in Fig 3a and 3b; a decline in grip strength is observed for girls after 13 years, while in boys a linear increase occurs from 12 years onwards.

### The shuttle-run speed test

The multiplicative model relating the children’s shuttle run speed (note that we transformed the run time into speed (m∙s^-1^), i.e., total distance run (50m)/ time in seconds) to their body size components and maturity offset estimate was found to be:

$$\text{Shuttle run speed }(\text{m }\cdot \;{\text{s}}^{-1})\;=\text{ a}\cdot \;{\text{mass}}^{-0.105}\;\cdot \;{\text{height}}^{0.177}\;\cdot \text{ exp}(0.010\;\cdot \text{ maturity offset})$$ (6)

**Fig 4:**
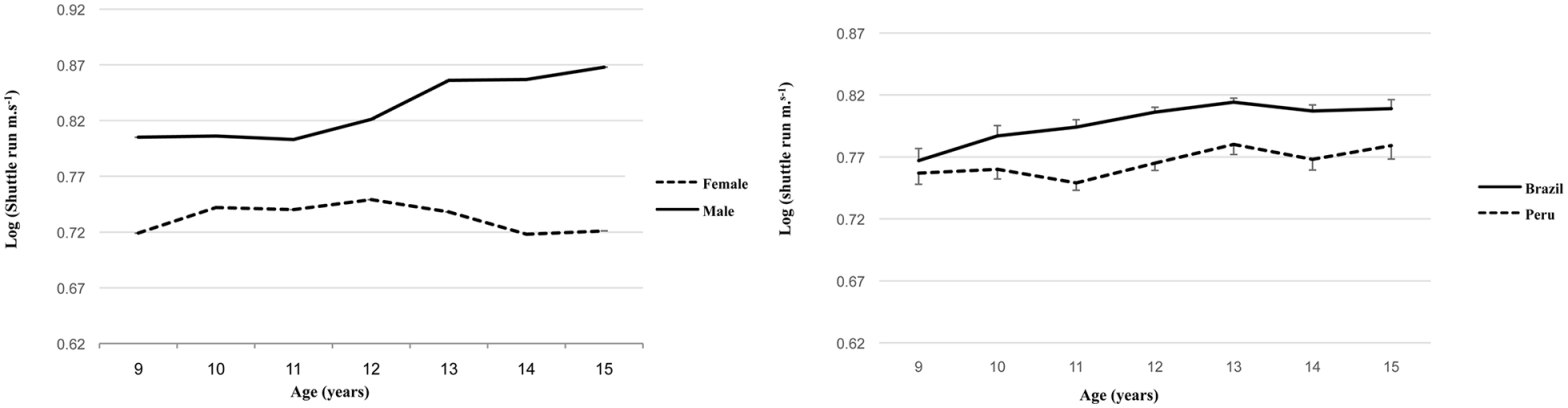
Trends in means (logs of shuttle run) in all boys and girls, irrespective of country, across age (left), and between Peruvians and Brazilians, irrespective of sex, across age (right).

The mass and height exponents were k_1_ = -0.105 (SEE = 0.011), and k_2_ = 0.177 (SEE = 0.049), with a significant maturation offset parameter b = 0.010 (SEE = 0.005). As with the 12-minute run test, the height and mass exponents have opposite signs suggesting that a height-to-weight ratio, not too dissimilar to inverse BMI [43], is the optimal body shape associated with the 12-minute run test. The adj R^2^ was 28.3%, but no significant effects due to leisure time PA and sports participation (p = 0.711 and 0.118), respectively were found, having controlled for differences in mass, height, sex, age and country. The constant *“a”* varied significantly with the main effects for sex, maturity, age, and country and all interactions between these categorical variables (p<0.001). From adjusted marginal means, their anti-logs and differences calculated between the two samples, Brazilian children and youth were, on average, ~1.0 s faster than Peruvians. Boys are also, on average, ~2.2 s faster than girls. The interactions between sex, age and country in shuttle speed (<0.001) are plotted in Fig 4a and 4b; it is noteworthy that girls decline their speed from 12 years onwards, while boys increase their performance in shuttle run from 11 years onwards.

## Discussion

An allometric modelling approach was used to identify optimal body size and shape characteristics associated with MP. In general, Brazilian boys and girls outperformed Peruvians at all ages not only in absolute terms, but also when adjusting for body size/shape, biological maturation and PA. Boys also outperformed girls within each country, even when controlling for body size and shape.

In the 12-minute run test it was found that height-to-body mass ratio (known as the RPI = height/mass^0.333^) best characterizes the optimal relationship between running performance and shape, i.e., boys and girls who are relatively taller displaying a linear physique, mostly ectomorphic, excel in this test. Analogous findings were reported by Nhantumbo et al. [26], and Bustamante et al. [27] with similar height-to-body mass ratios in Mozambican and Peruvian children and adolescents, respectively, as well as by Nevill et al. [6] with Greek children. Physical activity was not significantly associated with Brazilians and Peruvians’ 12-minute run, although it has been recognized that organized PA will enhance aerobic fitness with various effect sizes [8]. Yet, the systematic positive relationship between youngsters’ current PA levels and aerobic fitness remains to be proven [44].

It has been consistently reported that during childhood and adolescence the absolute values of the aerobic fitness components vary in relation to age, biological maturation and sex [45]. Given that the maturity offset parameter was not statistically significant, biological maturation does not seem to be an important correlate of the results of this test, even after adjusting for body size. On the contrary, Beunen et al. [46] observed that VO_2_ peak was largely explained by body mass, and that physical activity and its interaction with maturity status contributed independently to VO_2_ peak. Further, when aerobic performance was usually expressed in relative terms (ml∙kg^-1^∙min^-1^) Beunen et al. [46] did not find systematic age differences in boys, while a decline was observed in girls, a common result observed in previous reports [8,12,47]. Further, marked sex differences in youth cardiorespiratory fitness are well-known when absolute values are considered and it was also observed in our data. In a recent review, Olds et al. [1] showed consistent differences between sexes, in every age group, across a wide range of countries. Additionally, given that Peruvian children and adolescents are taller and heavier than Brazilians, their 12-min run performance was expected to be lower indicating that heavier children tend to be worse performers in running and jumping tasks [48].

The allometric models used to predict explosive power (standing long jump) and static strength (handgrip) identified different optimal height-to-body mass ratios mostly linked to motor tasks’ specificities. In the standing long jump, boys and girls with linear physiques have advantages since the body needs to be horizontally propelled; on the contrary, in the handgrip, the mass and height exponents are both positive, suggesting that both greater mass (probably muscle mass) and greater height (probably reflecting greater leverage) are required to display higher grip strength values. Biological maturation and PA were positively associated with these strength markers reflecting their probable interactions with body size in optimizing adolescent’s MP [12]. These are expected results since early maturing girls tend to be stronger and taller than later maturing girls [12]. It has been shown that the associations between maturity and strength are positive during the adolescent period [12,49,50]. For example, early maturing Canadian boys [51] had significantly greater strength than late maturers; further, in a longitudinal study with Canadian girls aged 10 to 14 years followed consecutively for 3 years, Little et al. [52] reported significant improvements in their explosive and static strength with advancing age and sexual maturation. In the present study, sex differences were identified not only in absolute terms but also when controlling for body size/shape. These sex-differences can be partly attributable to differences in total muscle mass induced by hormonal changes, namely in testosterone levels linked with the onset of puberty and the growth spurt in boys [12]. When we contrasted countries, Brazilians where, on average, shorter than Peruvians but outperformed them. Analogous results were shown by Bustamante et al. [27] with adolescent boys and girls from three different Peruvian geographical regions, as well as by Santos [53] in a cross-cultural study between Mozambican and Portuguese children/adolescents.

In the shuttle run test, the allometric model showed negative body mass and positive height exponents, suggesting that an appropriate proportion of height/body mass is connected with greater performance. Further, those advanced in their biological maturation status benefited most in their performance, which is consistent with Malina et al. [12] suggestion about early teenage maturers’ (ahead in their PHV years) overall motor performance. As verified in the previous tests, sex-differences are also present. Girls reduced their speed from 12 years onwards, while boys increased theirs from 11 years onwards. Nhantumbo et al. [26] and Bustamante et al. [27] found similar results with Mozambican and Peruvian and adolescents, respectively.

Brazilian schoolchildren are better performers than Peruvians at all ages. As is well-known, agility refers to the ability to change the direction of the body abruptly or to shift quickly the direction of movement. Okely et al. [54] reported that agility is dependent on a suitable combination of factors such as speed, strength, and balance; further, Malina et al. [12] suggested that fat mass negatively impacts on some domains of physical performance and overall motor functioning, while lean mass is less significant in absolute terms but is important relative to amount of body fat. In a previous study, Bustamante and Maia [55] found differences in MP and nutritional status between Barranco schoolchildren and others from different Peruvian regions, i.e., Barranco children’s had higher adiposity and lower PA levels. It is important to emphasize that Barranco is a district that presents a fully urbanized small geographic area, with a high population density, serious problems of traffic congestion and of public insecurity, besides a lack of infrastructure for recreational and sporting activities on and off the school [56]. These conditions are unfavorable for Barranco school children to adopt healthy and active lifestyles; moreover, they have fewer opportunities given their environmental constraints for developing their physical fitness and other motor kills, which in turn may also be associated with their higher prevalence of overweight and obesity than their peers from other Peruvian regions [55].

Notwithstanding the importance of the present data in terms of their cross-cultural perspectives, three limitations have to be acknowledged. The first relates to country representativeness in terms of sample size and diversity. As such, we caution readers to have this fact in mind when trying to generalize our findings. Yet, this study represent an attempt to properly interpret children and adolescents´ motor performance expressed by different tests especially when their body size differs as well as their geographic and socioeconomic environmental conditions. The second limitation relates to the way in which biological maturation was estimated, as the Mirwald et al. [34] equations have not been cross-validated in Brazilian and Peruvian children and adolescents. The degree to which this introduced bias in the results is not known. However, all markers of biological maturity have associated errors and problems with using them in the cross-cultural context. In addition, the use of x-rays or the assessment of secondary sexual maturation characteristics would not have been feasible in this study. The third one relates to the fact that PA was estimated via questionnaire which is prone to several well-known limitations. Yet, given the large sample size and limited resources available, we were left with no other instruments to proceed otherwise. In order to minimize errors in responding to the questionnaire, children and adolescents completed them during their physical education classes with the consistent help of their physical education teachers and research team members. This report also has two strengths. First, the relatively large sample size covering childhood and puberty periods. Second, the cross-cultural nature including youth from two developing countries living in different environmental contexts.

## Conclusions

In summary, the current study identified the key body shape associated with four body mass-dependent MP tests, which parallels the known RPI (height^3^/body mass or height/body mass^0.333^). Further, biological maturation is mostly relevant in strength, power and agility tasks, and PA is also positively associated with strength performance. Sex-differences were found in all motor tests, as well as across countries favoring Brazilian children, even when accounting for their body size/shape differences. Finally, the use of allometric models with age, sex, maturity, and physical activity levels as covariates of log-transformed height and body mass provided valid inference to analyze motor performance differences between the schoolchildren from different populations.
